# A systematic review and meta-analysis of loratadine combined with montelukast for the treatment of allergic rhinitis

**DOI:** 10.3389/fphar.2023.1287320

**Published:** 2023-10-17

**Authors:** Huan Wang, Qing Ji, Chao Liao, Li Tian

**Affiliations:** ^1^ Hospital of Chengdu University of Traditional Chinese Medicine, Chengdu, Sichuan, China; ^2^ Chengdu University of Traditional Chinese Medicine, Chengdu, Sichuan, China; ^3^ Chengdu First People’s Hospital, Chengdu, Sichuan Province, China

**Keywords:** allergic rhinitis, meta-analysis, loratadine, montelukast, combination therapy

## Abstract

**Background:** Loratadine and montelukast are clinical first-line drugs in the treatment of allergic rhinitis (AR). However, there is no clear evidence of the efficacy of loratadine combined with montelukast in the treatment of AR. This study aimed to evaluate the efficacy and safety of the loratadine-montelukast combination on AR.

**Methods:** In this meta-analysis, searches were conducted on PubMed, Embase, the Cochrane Central Register of Controlled Trials, Web of Science, and China National Knowledge Infrastructure (CNKI). The search terms included loratadine, montelukast, allergic rhinitis, and clinical trials. Meta-analyses were conducted using Rev Man 5.3 and Stata 15 statistical software.

**Results:** A total of 23 studies with 4,902 participants were enrolled. For the primary outcome, pooled results showed that loratadine-montelukast can significantly reduce total nasal symptom scores (TNSS), when compared with loratadine (SMD, −1.00; 95% CI, −1.35 to −0.65, *p* < 0.00001), montelukast (SMD, −0.46; 95% CI, −0.68 to −0.25, *p* < 0.0001), or placebo (SMD, −0.93; 95% CI, −1.37 to −0.49, *p* < 0.00001). For secondary outcomes, pooled results showed that compared with loratadine, loratadine-montelukast can significantly improve nasal congestion, nasal itching, nasal sneezing, nasal rhinorrhea, and rhinoconjunctivitis quality of life questionnaires (RQLQ). Compared with montelukast, loratadine-montelukast can significantly improve nasal itching, and nasal sneezing. Compared with placebo, loratadine-montelukast can significantly improve nasal congestion, and RQLQ.

**Conclusion:** Loratadine-montelukast combination is superior to loratadine monotherapy, montelukast monotherapy, or placebo in improving AR symptoms. Therefore, loratadine-montelukast combination can be an option for patients with moderate-severe AR or poorly response to monotherapy.

Systematic review registration number: clinicaltrials.gov, identifier CRD42023397519.

## 1 Introduction

Allergic rhinitis (AR) is an allergic disease and is the sixth most prevalent chronic disease globally (Bousquet et al., 2020; Maladkar, 2023). The incidence and recurrence rate of AR is extremely high, and up to 40% of the population worldwide is affected by AR (Cheng et al., 2018). It has become a global health problem that cannot be ignored. AR consumes huge medical resources and causes a huge economic burden to individuals and society. At the same time, it also affects people’s sleep, quality of life, and physical and mental health. Research shows that AR patients have associated anxiety and depression (Rodrigues et al., 2021; Rodrigues et al., 2022), especially in women (Bedolla-Barajas et al., 2017).

The pathophysiology of AR is complex, involving an early and late phase responses. The main feature of the early phase response is mast cell degranulation, which occurs within minutes of allergic individuals being exposed to the allergen and lasts for approximately 2–3 h. When the body is exposed to an allergen, a specific immune response occurs and IgE antibodies are produced (Bernstein, Schwartz, and Bernstein, 2016). When the same allergen is inhaled, the specific allergen cross-links with allergen specific IgE bound to the mast cell surface, and the mast cells degranulate and release various preformed and newly formed mediators, such as histamine (Pawankar et al., 2011). Histamine stimulates the sensory nerve endings of the trigeminal nerve and induces sneezing (Naclerio, 1991). The critical role of type 1 histamine receptor (H1R) in histamine-induced neuronal symptoms has been demonstrated by suppression of sneezing, nasal itching, and rhinorrhea by H1R antagonists following histamine nasal stimulation (Hilberg, Grymer, and Pedersen, 1995; Wood-Baker, Lau, and Howarth, 1996; Wang et al., 2001). H1R antagonists, however, have been far less effective in reducing allergen-induced nasal obstruction. The late phase response usually occurs 4–6 h after allergen challenge, with symptoms lasting 18–24 h. The key to the late phase response is the release of cytokines and chemokines from mast cells, such as IL-4, IL-13 (Bradding et al., 1993; Pawankar et al., 1995; Pawankar and Ra, 1996). These cytokines can upregulate the expression of vascular cell adhesion factors on endothelial cells, making it easier for T lymphocytes, neutrophils, and eosinophils to infiltrate nasal mucosal tissue and cause inflammatory reactions. The late phase response is characterized by the influx of inflammatory cells and the release of inflammatory mediators such as leukotrienes, kinins, and histamine. Cysteinyl leukotrienes (CysLTs) play a major role in AR. Among them, CysLT1 receptor is a high-affinity leukotriene D4 receptor that is sensitive to CysLT1 receptor antagonists currently used to treat asthma and AR. CysLT1 antagonists have been shown to attenuate antigen-induced nasal airway resistance and increase nasal vascular permeability. Studies have shown that CysLT1 receptor antagonists are as effective as antihistamines in treating AR and may be useful in treating upper and lower respiratory tract allergic diseases (Shirasaki and Himi, 2016). In addition, other mediators, such as eosinophil cationic protein (ECP), platelet-activating factor, and major basic protein (MBP), are also involved in the late phase response.

The current AR drug therapy includes antihistamines, cysteinyl leukotriene receptor antagonists, intranasal corticosteroids, mast cell stabilizers, anticholinergics, and decongestants. However, with monotherapy it is hard to achieve complete symptom resolution of AR. A survey demonstrated that a large proportion of patients with AR are not satisfied with their treatment, and up to 60% of patients have an interest in finding new management for AR (Nathan, 2007). Therefore, the combination of these monotherapies is proposed to yield additive therapeutic effects since multiple inflammatory pathways were targeted. Loratadine, a histamine H1-receptor antagonist, and montelukast, a cysteinyl leukotriene receptor antagonist, are main drugs for AR therapy. However, the effect of concomitant montelukast and loratadine on AR is remain unclear, given there has not been a published meta-analysis on this topic. We performed this systematic review and meta-analysis to study the effect of the combined application of these two drugs on the treatment of AR symptoms to provide an evidence-based reference for rational drug use in the clinic.

## 2 Methods

### 2.1 Methods and search strategy

The systematic review was conducted and reported in accordance to Preferred Reporting Item for Systematic Reviews and Meta-analysis (Moher et al., 2009). The study protocol was registered in the International Prospective Register of Systematic Reviews; registration number: CRD42023397519. Studies were searched in the following databases: PubMed database, Embase, the Cochrane Central Register of Controlled Trials, Web of Science, and China National Knowledge Infrastructure (CNKI). The search keywords included the Medical Subject Headings terms “loratadine”, “montelukast”, “allergic rhinitis”, and “randomized controlled trial”. The search was limited to studies performed on humans. The language of the searched articles is limited to English and Chinese. The details of the search strategy and key words search in PubMed database were taken as an example and presented in Supplementary Table S1. In addition, clinical trial registries, reviews and references of similar clinical studies were also reviewed to search for potentially relevant studies.

### 2.2 Data sources and study selection

Two independent reviewers evaluated the titles and abstracts and obtained the full-text articles of the relevant trials. Differences of opinion were resolved by consensus between the reviewers, and if necessary, by consulting with other reviewers. The full-text articles of the selected randomized controlled trials (RCTs) were reviewed.

### 2.3 Inclusion and exclusion criteria

Inclusion criteria include as followings: 1) The study should be RCTs 2) The study compared the effect of combination treatment (loratadine-montelukast) with loratadine, montelukast or placebo in patients with AR. 3) The study includes primary outcomes or secondary outcomes. The primary outcome was total nasal symptom score (TNSS). The secondary outcomes were single nasal symptom scores including nasal congestion score (NCS), nasal itching score (NIS), sneezing score, and rhinorrhea score, and rhinoconjunctivitis quality of life questionnaires (RQLQ). The trials were excluded if they were 1) non-human studies, 2) non-comparative studies, 3) studies with data that could not be extracted, 4) non-original studies (letters, reviews, comments), 5) case reports, 6) non-AR, 7) and ongoing trials without results.

### 2.4 Data extraction and quality assessment

Two independent reviewers extracted trial characteristics (first name of author, year of publication, country, design of study, study duration), patient baseline characteristics (age, gender, type of patients), intervention description (intervention strategy), results and adverse events. The Cochrane Handbook was used to assess quality of the included trials. Quality assessment included selection bias, performance bias, detection bias, attrition bias, reporting bias, and other sources of bias. The risk of bias was assessed by two independent reviewers. The third author resolved the discrepancies. The corresponding author was responsible for contacting authors of trials to obtain missing information and unpublished data.

### 2.5 Data synthesis and analysis

The pooled results are displayed as standardized mean difference (SMD), and 95% confidence interval (CI). When the combination group compared with more than one control groups, the number of patients in the combination group was divided by the number of the control groups. Heterogeneity was reported by I^2^, and I^2^ values < 25%, between 25% and 50%, and >50% were defined as a low, moderate, and high degree of heterogeneity, respectively (Higgins et al., 2003). The fixed model was used in low or moderate heterogeneity, and the random model was used in significant heterogeneity. A funnel plot asymmetry test was performed when a result included more than 10 trials. Sensitivity analysis was performed by deleting one study at a time and recalculating the pooled result to detect the robustness of the overall results. *p*-value <0.05 is considered statistically significant. Subgroup analysis was conducted based on whether the studies were published in Chinese or English, as well as on whether the participants were adults or children. Cochrane Review Manager 5.3 (Oxford, UK) and Stata 15 software was used for the meta-analysis.

## 3 Results

### 3.1 Study selection, and characteristics

**FIGURE 1:**
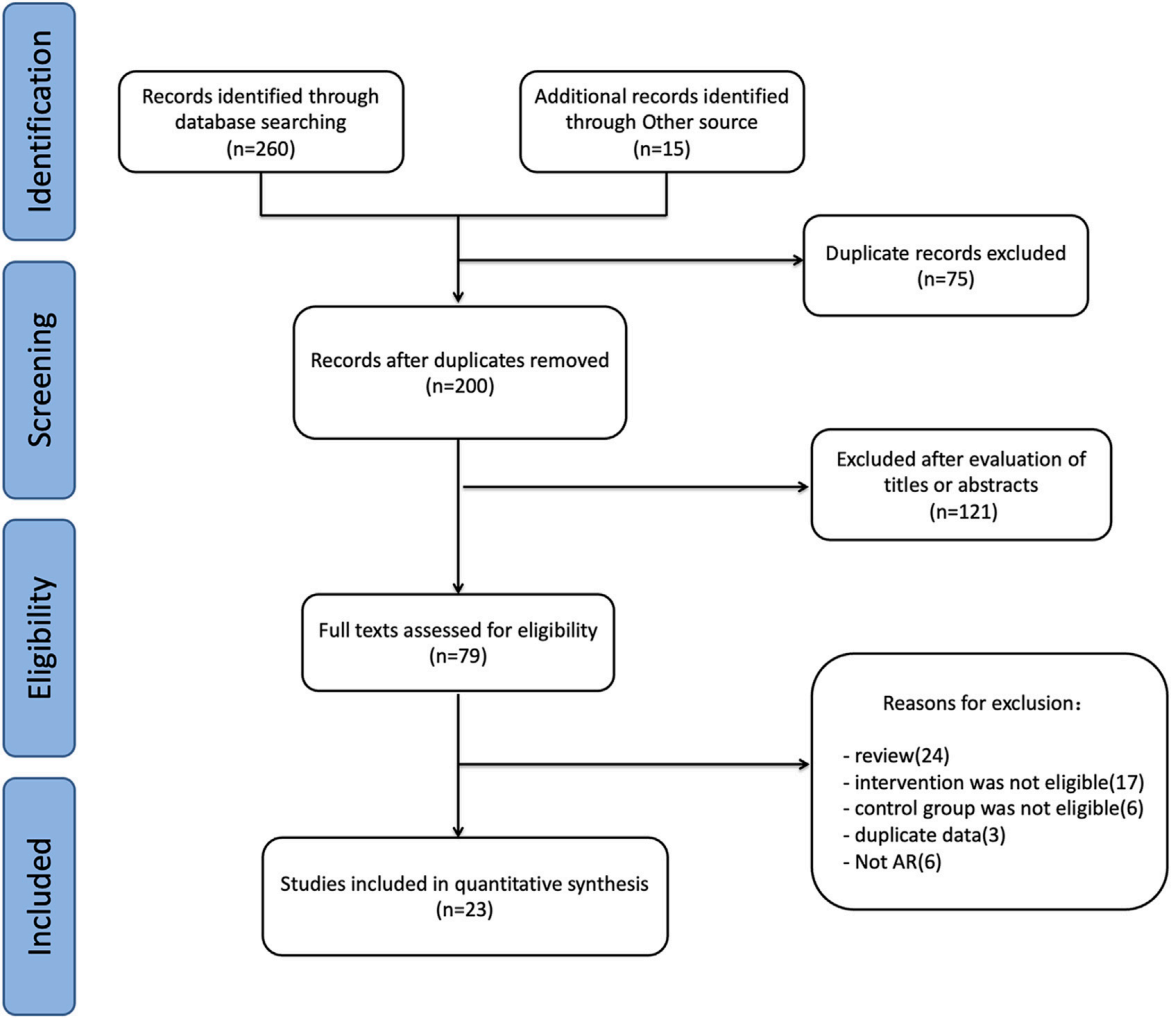
Studies selection flow for the meta-analysis review.

There were 275 relevant studies identified through the initial search, 41 were from PubMed, 44 were from Embase, 31 were from Web of Science, 72 were from Cochrane Library, 8 were from Clinical Trials, 64 were from CNKI, and 15 articles were from other sources. After excluding duplicate articles and further evaluating the full text of the remaining articles, 23 articles (Meltzer et al., 2000; Nayak et al., 2002; Pullerits et al., 2002; Hung et al., 2007; Xiao and Zhang, 2008; Day et al., 2009; Huang et al., 2009; Lu et al., 2009; Prenner et al., 2009; Horak et al., 2010; Yamamoto et al., 2012; Gong, 2016; Cai et al., 2017; Lin, 2017; Bian and Cui, 2018; Li, 2019; Liu, Zeng, and Zeng, 2019; Miao, 2020; Qiao, 2020; Shen et al., 2020; Zhang et al., 2021; Lu et al., 2022; Shi et al., 2022) were finally included in this meta-analysis. Lu et al. (Lu et al., 2009) reported 2 trials, which were identified as Lu a, and Lu b. Meltzer et al. (Meltzer et al., 2000) compared two doses of montelukast at 10 mg and 20 mg with loratadine-montelukast simultaneously, which were identified as Meltzer a, and Meltzer b. Shen et al. (Shen et al., 2020) divided the subjects into a sneezing group, a nasal congestion group and a mixed group according to different scores of nasal congestion and sneezing, which were identified as Shen a, Shen b, and Shen c. The flow diagram of study selection is presented in Figure 1.

### 3.2 Participants

A total of 23 studies with 24 trials (Meltzer et al., 2000; Nayak et al., 2002; Pullerits et al., 2002; Hung et al., 2007; Xiao and Zhang, 2008; Day et al., 2009; Huang et al., 2009; Lu et al., 2009; Prenner et al., 2009; Horak et al., 2010; Yamamoto et al., 2012; Gong, 2016; Cai et al., 2017; Lin, 2017; Bian and Cui, 2018; Li, 2019; Liu, Zeng, and Zeng, 2019; Miao, 2020; Qiao, 2020; Shen et al., 2020; Zhang et al., 2021; Lu et al., 2022; Shi et al., 2022) and 4,902 participants were enrolled. 1976 patients in all studies were treated with loratadine-montelukast, 1,264 in 18 trials were with loratadine (Meltzer et al., 2000; Nayak et al., 2002; Hung et al., 2007; Xiao and Zhang, 2008; Huang et al., 2009; Lu et al., 2009; Gong, 2016; Cai et al., 2017; Lin, 2017; Bian and Cui, 2018; Li, 2019; Liu, Zeng, and Zeng, 2019; Qiao, 2020; Shen et al., 2020; Zhang et al., 2021; Lu et al., 2022; Shi et al., 2022), 768 in 10 trials with montelukast (Meltzer et al., 2000; Nayak et al., 2002; Pullerits et al., 2002; Xiao and Zhang, 2008; Huang et al., 2009; Lu et al., 2009; Yamamoto et al., 2012; Miao, 2020; Shen et al., 2020), and 894 in 8 trials with placebo (Meltzer et al., 2000; Nayak et al., 2002; Pullerits et al., 2002; Day et al., 2009; Lu et al., 2009; Prenner et al., 2009; Horak et al., 2010). 5 RCTs trials included pediatric patients with AR (Hung et al., 2007; Xiao and Zhang, 2008; Huang et al., 2009; Cai et al., 2017; Lu et al., 2022), and the remaining 19 trials included adults AR (Meltzer et al., 2000; Nayak et al., 2002; Pullerits et al., 2002; Day et al., 2009; Lu et al., 2009; Prenner et al., 2009; Horak et al., 2010; Yamamoto et al., 2012; Gong, 2016; Lin, 2017; Bian and Cui, 2018; Li, 2019; Liu, Zeng, and Zeng, 2019; Miao, 2020; Qiao, 2020; Shen et al., 2020; Zhang et al., 2021; Shi et al., 2022). 1 trial (Lu et al., 2009) included patients with AR and comorbid asthma. 10 studies were English articles (Meltzer et al., 2000; Nayak et al., 2002; Pullerits et al., 2002; Hung et al., 2007; Day et al., 2009; Lu et al., 2009; Prenner et al., 2009; Horak et al., 2010; Yamamoto et al., 2012; Shen et al., 2020), and the remaining 13 were Chinese articles (Xiao and Zhang, 2008; Huang et al., 2009; Gong, 2016; Cai et al., 2017; Lin, 2017; Bian and Cui, 2018; Li, 2019; Liu, Zeng, and Zeng, 2019; Miao, 2020; Qiao, 2020; Zhang et al., 2021; Lu et al., 2022; Shi et al., 2022). The duration of intervention administration varied between studies and ranged from 4 hours to 2 months. The most frequent outcome measures were TNSS, and nasal congestion symptom score. The characteristics of these 23 studies are shown in Table 1.

**TABLE 1:** Basic characteristics of subjects and treatments of trials.

Source	Country	Type of patients	Duration	Intervention			Control			Outcomes	Adverse event
				Intervention method (Dose)	Population (Male)	Mean age (years ±SD)	Intervention method (Dose)	Population (Male)	Mean age (years ±SD)		
Bian and Cui (2018)	China	AR	2 weeks	Loratadine-montelukast (10mg–4mg, once/day)	65 (40)	37.4 ± 11.5	Loratadine (10mg, once/day)	65 (42)	37.5 ± 11.6	TNSS	Headache, Dry mouth, Drowsiness
Cai et al. (2017)	China	AR in children	2 weeks	Loratadine-montelukast (10/5mg-5/4mg, once/day)	38 (20)	5.9 ± 2.1	Loratadine (10/5mg, once/day)	38 (21)	5.5 ± 2.4	VAS, Nasal Congestion score	Not reported
Day et al., 2009	Canada	SAR	8 h	Loratadine-montelukast (10mg–10mg, once/day)	127 (49)	33.6 ± 11.3	Placebo (once/day)	126 (51)	33.4 ± 10.6	TNSS, Nasal Congestion score	abdominal distention, abdominal pain, epigastric discomfort, headache, somnolence, nausea, hypoesthesia, disorientation, urticaria
Gong KL 2016	China	AR	2 weeks	Loratadine-montelukast (10mg–4mg, once/day)	42 (30)	40.34 ± 3.29	Loratadine (10 mg, once/day)	41 (29)	40.13 ± 3.02	TNSS RQLQ	Headache dry mouth drowsiness
Horak et al., 2010	Austria	SAR	4 h	Loratadine-montelukast (10mg–10 mg)	37 (17)	25	Placebo	37 (15)	26.5	TNSS, Nasal Congestion score	mild upper respiratory tract infection, severe knee fracture
Huang et al., 2009	China	AR in children	3 weeks	Loratadine-montelukast (10/5mg-5/4mg, once/day)	50 (27)	9.45 ± 3.97	Loratadine (10/5 mg, once/day)	50 (24)	9.23 ± 4.46	TNSS	Not reported
							montelukast (5/4mg, once/day)	50 (28)	9.13 ± 3.78		
Hung et al., 2007	China Taiwan	PAR in children	8weeks	Loratadine-montelukast (5mg–5 mg)	11 6)	8.45 ± 1.51	Loratadine (5 mg)	11 5)	7.81 ± 1.94	TNSS	Not reported
Li 2019	China	AR	2 weeks	Loratadine-montelukast (10mg–4mg, once/day)	40 (23)	43.51 ± 8.37	Loratadine (10mg, once/day)	40 (24)	43.46 ± 8.64	TNSS	headache, dry mouth, drowsiness
Lin 2017	China	AR	2 weeks	Loratadine-montelukast (10mg–4mg, once/day)	40 (19)	36.4 ± 2.8	Loratadine (10mg, once/day)	40 (22)	37.2 ± 2.5	TNSS RQLQ	headache, dry mouth, drowsiness
Liu et al., 2019	China	AR	1 month	Loratadine-montelukast (8.8mg–10mg, once/day)	52 (24)	Not reported	Loratadine (8.8mg, once/day)	52 (27)	Not reported	VAS Nasal Congestion, Itching, Sneezing, Rhinorrhea score	No adverse reactions
Lu et al., 2009	United States	SAR	2 weeks	Loratadine-montelukast (10mg–10 mg)	174	34.0 ± 12.7	Loratadine (10 mg)	115	34.8 ± 12.4	TNSS	Not reported
							Montelukast (10 mg)	111	35.6 ± 13.1		
							Placebo	57	35.1 ± 13.8		
		SAR and asthma	2 weeks	Loratadine-montelukast (10mg–10 mg)	209	32.8 ± 12.6	Loratadine (10 mg)	162	30.6 ± 10.9	TNSS	Not reported
							Montelukast (10 mg)	103	31.1 ± 13.1		
							Placebo	53	33.6 ± 13.5		
Lu et al., 2022	China	AR in children	3 months	Loratadine-montelukast + triamcinolone acetonide (10/5mg–5mg, once/day)	42 (26)	11.19 ± 2.12	Loratadine + triamcinolone acetonide (10/5mg, once/day)	42 (23)	11.81 ± 2.18	Nasal Congestion, Itching, Sneezing, Rhinorrhea score	Not reported
Meltzer et al., 2000	United States	SAR	2 weeks	Loratadine-montelukast (10mg–10 mg)	90 (44)	37	Loratadine (10 mg)	92 (43)	34.5	TNSS, Nasal Congestion, Itching, Sneezing, Rhinorrhea score, RQLQ	Headache, upper respiratory tract infection
							Montelukast (10 mg)	95 (40)	33		
							Montelukast (20 mg)	90 (33)	34.5		
							Placebo	91 (45)	33		
Miao 2020	China	AR	2 weeks	Loratadine-montelukast (4mg–10mg, once/day)	40 (25)	36.49 ± 4.52	Montelukast (10mg, once/day)	40 (24)	36.58 ± 4.71	TNSS RQLQ	Not reported
Nayak et al., 2002	United States	SAR	2 weeks	Loratadine-montelukast (10mg–10 mg)	302 (94)	38 ± 13	Loratadine (10 mg)	301 (110)	37 ± 13	TNSS, Nasal Congestion, Itching, Sneezing, Rhinorrhea score, RQLQ	Headache, dry mouth, asthenia, fatigue, tachycardia, prurit
							Montelukast (10 mg)	155 (53)	35 ± 11		
							Placebo	149 (63)	37 ± 13		
Prenner et al., 2009	United States	SAR	15 days	Loratadine-montelukast (10mg–10 mg)	363 (126)	26 ± 7	Placebo	363 (144)	22 ± 6	Nasal Congestion score, RQLQ	Dry mouth, Nausea, Headache, Insomnia, Vertigo, Irritability, Psychomotor hyperactivity, Tremor, Nervousness, Restlessness
Pullerits et al., 2002	Sweden	SAR	2 months	Loratadine-montelukast (10mg–10 mg)	15 6)	30.1 ± 9.9	Montelukast (10 mg)	16 (10)	28.3 ± 8.0	TNSS	Not reported
							Placebo	18 (13)	29.8 ± 10.4		
Qiao 2020	China	AR	2 weeks	Loratadine-montelukast (8.8mg–10mg, once/day)	51 (30)	42.39 ± 5.46	Loratadine (8.8mg, once/day)	51 (28)	42.57 ± 5.53	Nasal Congestion, Itching, Sneezing, Rhinorrhea score	dry mouth, drowsiness, dizziness, rash
Shen et al., 2020	China	Patients with AR in sneezing group	8 weeks	Loratadine-montelukast + mometasone furoate (10 mg/10 mg)	9	Not reported	Loratadine + mometasone furoate	8	Not reported	TNSS	Not reported
							montelukast + mometasone furoate	8			
		Patients with AR in nasal congestion group	8 weeks	Loratadine-montelukast + mometasone furoate (10 mg/10 mg)	10	Not reported	Loratadine + mometasone furoate	9	Not reported	TNSS	Not reported
							montelukast + mometasone furoate	9			
		Patients with AR in sneezing and nasal congestion group	8 weeks	Loratadine-montelukast + mometasone furoate (10 mg/10 mg)	10	Not reported	Loratadine + mometasone furoate	9	Not reported	TNSS	Not reported
							montelukast + mometasone furoate	10			
Shi et al., 2022	China	AR	2 weeks	Loratadine-montelukast (10mg–10mg, once/day)	37 (21)	44.23 ± 3.18	Loratadine (10mg, once/day)	37 (22)	43.96 ± 3.05	Nasal Congestion, Itching, Sneezing, Rhinorrhea score	diarrhea, rash, dizziness, headache, nausea and vomiting
Xiao and Zhang (2008)	China	AR in children	2 weeks	Loratadine-montelukast (10/5mg-5/4mg, once/day)	60 (32)	8.52 ± 3.2	Loratadine (10/5mg, once/day)	60 (28)	8.45 ± 3.27	TNSS	Not reported
							montelukast (5/4mg, once/day)	60 (35)	8.33 ± 3.16		
Yamamoto et al., 2012	Japan	SAR	50 days	Loratadine-montelukast (10mg–10 mg)	21 7)	26.7 ± 2.4	Montelukast-placebo (10 mg)	21 (10)	26.4 ± 2.2	TNSS	Not reported
Zhang et al., 2021	China	AR	2 weeks	Loratadine-montelukast (10mg–5mg, once/day)	41 (23)	41.2 ± 4.59	Loratadine (10mg, once/day)	41 (24)	42.16 ± 4.72	Nasal Congestion, Itching, Sneezing, Rhinorrhea score	headache, dry mouth, drowsiness

RCT: randomized controlled trial; AR: allergic rhinitis; TNSS: total nasal symptom scores; SAR: seasonal allergic rhinitis; PAR: perennial allergic rhinitis; RQLQ: rhinoconjunctivitis quality of life questionnaires; VAS: visual analogue scale.

### 3.3 Intervention

11 trials compared loratadine-montelukast with loratadine (Hung et al., 2007; Gong, 2016; Cai et al., 2017; Lin, 2017; Bian and Cui, 2018; Li, 2019; Liu, Zeng, and Zeng, 2019; Qiao, 2020; Zhang et al., 2021; Lu et al., 2022; Shi et al., 2022), 2 trials compared loratadine-montelukast with montelukast (Yamamoto et al., 2012; Miao, 2020), 3 trials compared loratadine-montelukast with placebo (Day et al. 2009; Prenner et al. 2009; Horak et al. 2010), 3 trials compared loratadine-montelukast with loratadine and montelukast (Xiao and Zhang, 2008; Huang et al. 2009; Shen et al. 2020), 1 trial compared loratadine-montelukast with montelukast and placebo (Pullerits et al. 2002), 3 trials compared loratadine-montelukast with loratadine, montelukast, and placebo (Meltzer et al. 2000; Nayak et al., 2002; Lu et al., 2009). Loratadine, and montelukast were administered orally in all studies. The dosage of loratadine was different in different studies. Among them, the dosage of 16 trials were 10 mg (Meltzer et al., 2000; Nayak et al., 2002; Pullerits et al., 2002; Day et al., 2009; Lu et al., 2009; Prenner et al., 2009; Horak et al., 2010; Yamamoto et al., 2012; Gong, 2016; Lin, 2017; Bian and Cui, 2018; Li, 2019; Shen et al., 2020; Zhang et al., 2021; Shi et al., 2022), 1 trial was 5 mg (Hung et al., 2007), 1 trial was 4 mg (Miao, 2020), 2 trials were 8.8 mg (Liu, Zeng, and Zeng, 2019; Qiao, 2020), and 4 trials were 10 or 5 mg (Xiao and Zhang, 2008; Huang et al., 2009; Cai et al., 2017; Lu et al., 2022). The dosage of loratadine in children with AR was calculated according to body weight. For children <30 kg, the dose used was 5 mg and for children >30 kg, the dose was 10 mg. The dosage of montelukast was reported as followings: 1 trial was 20 mg (Meltzer et al., 2000), 14 trials were 10 mg (Meltzer et al., 2000; Nayak et al., 2002; Pullerits et al., 2002; Day et al., 2009; Lu et al., 2009; Prenner et al., 2009; Horak et al., 2010; Yamamoto et al., 2012; Liu, Zeng, and Zeng, 2019; Miao, 2020; Qiao, 2020; Shen et al., 2020; Shi et al., 2022), 3 trials were 5mg (Hung et al. 2007; Zhang et al. 2021; Lu et al. 2022), 4 trials were 4mg (Gong, 2016; Lin, 2017; Bian and Cui, 2018; Li, 2019), and 3 trials were 5 or 4mg (Xiao and Zhang, 2008; Huang et al. 2009; Cai et al., 2017). For children <6 years of age, montelukast 4 mg was administered while those >6 years of age received montelukast 5 mg.

### 3.4 Outcomes

Three comparisons, loratadine-montelukast *versus* loratadine, loratadine-montelukast *versus* montelukast, loratadine-montelukast *versus* placebo, were performed in this meta-analysis, and the primary and secondary outcomes were evaluated.

#### 3.4.1 Loratadine-montelukast vs. loratadine

##### 3.4.1.1 TNSS

**FIGURE 2:**
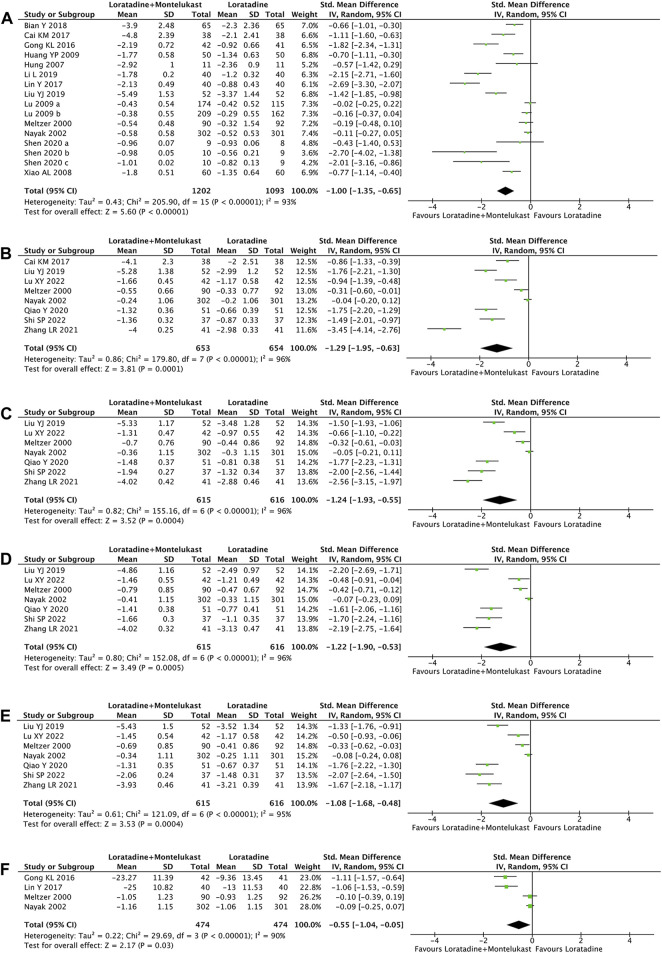
Forest plot for effect of loratadine-montelukast *versus* loratadine on total nasal symptom score **(A)**, nasal congestion **(B)**, nasal itching **(C)**, sneezing score **(D)**, rhinorrhea score **(E)**, and rhinoconjunctivitis quality-of-life questionnaires **(F)**.

Primary outcome was reported in 14 trials (Meltzer et al., 2000; Nayak et al., 2002; Hung et al., 2007; Xiao and Zhang, 2008; Huang et al., 2009; Lu et al., 2009; Gong, 2016; Cai et al., 2017; Lin, 2017; Bian and Cui, 2018; Li, 2019; Liu, Zeng, and Zeng, 2019; Shen et al., 2020). Compared with loratadine, loratadine-montelukast can significantly reduce TNSS (SMD, −1.00; 95% CI, −1.35 to −0.65, *p* < 0.00001, I^2^ = 93%, Figure 2A).

##### 3.4.1.2 Single nasal symptom score

7 trials reported NCS, NIS, sneezing score, and rhinorrhea score simultaneously (Meltzer et al., 2000; Nayak et al., 2002; Liu, Zeng, and Zeng, 2019; Qiao, 2020; Zhang et al., 2021; Lu et al., 2022; Shi et al., 2022), and 1 trial reported only the NCS (Cai et al., 2017). Compared with loratadine, loratadine-montelukast can significantly reduce nasal congestion (SMD, −1.29; 95% CI, −1.95 to −0.63, *p* < 0.0001, I^2^ = 96%, Figure 2B), nasal itching (SMD, −1.24; 95% CI, −1.93 to −0.55, *p* = 0.0004, I^2^ = 96%, Figure 2 C), sneezing (SMD, −1.22; 95% CI, −1.90 to −0.53, *p* = 0.0005, I^2^ = 96%, Figure 2 D), and rhinorrhea (SMD, −1.08; 95% CI, −1.68 to −0.48, *p* = 0.0004, I^2^ = 95%, Figure 2E).

##### 3.4.1.3 RQLQ

4 trials reported RQLQ (Meltzer et al., 2000; Nayak et al., 2002; Gong, 2016; Lin, 2017). This meta-analysis demonstrated a significant improvement in RQLQ (SMD, −0.55; 95% CI, −1.04 to −0.05, *p* = 0.03, I^2^ = 90%, Figure 2 F) in the loratadine-montelukast group compared with loratadine.

#### 3.4.2 Loratadine-montelukast vs. montelukast

##### 3.4.2.1 TNSS

**FIGURE 3:**
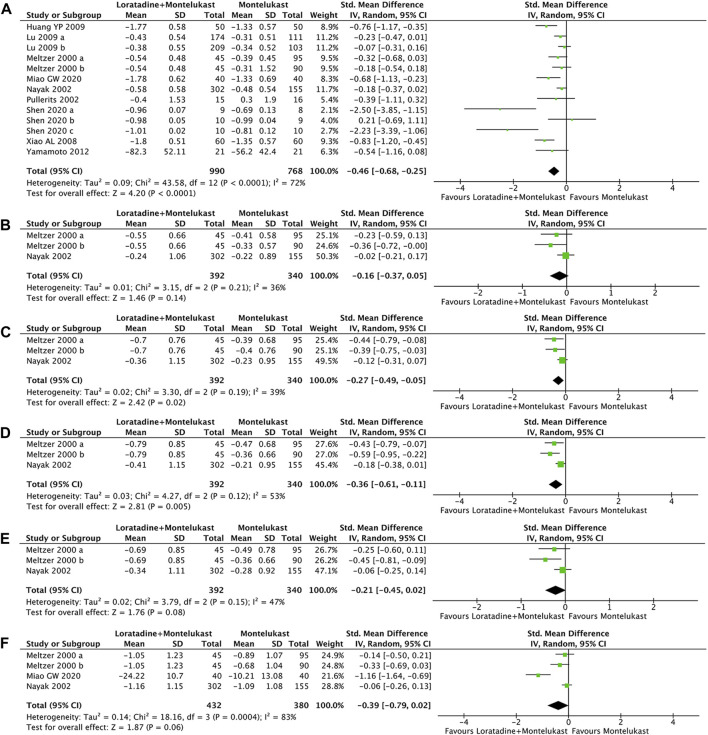
Forest plot for effect of loratadine-montelukast *versus* montelukast on total nasal symptom score **(A)**, nasal congestion **(B)**, nasal itching **(C)**, sneezing score **(D)**, rhinorrhea score **(E)**, and rhinoconjunctivitis quality-of-life questionnaires **(F)**.

Primary outcome was reported in 10 trials (Meltzer et al., 2000; Nayak et al., 2002; Pullerits et al., 2002; Xiao and Zhang, 2008; Huang et al., 2009; Lu et al., 2009; Yamamoto et al., 2012; Miao, 2020; Shen et al., 2020). Compared with montelukast, loratadine-montelukast can significantly reduce TNSS (SMD, −0.46; 95% CI, −0.68 to −0.25, *p* < 0.0001, I^2^ = 72%, Figure 3A).

##### 3.4.2.2 Single nasal symptom score

2 trials reported the four symptom scores of nasal congestion, nasal itching, sneezing, and rhinorrhea simultaneously (Meltzer et al., 2000; Nayak et al., 2002). Compared with montelukast, loratadine-montelukast can significantly reduce nasal itching (SMD, −0.27; 95% CI, −0.49 to −0.05, *p* = 0.02, I^2^ = 39%, Figure 3B), and sneezing (SMD, −0.36; 95% CI, −0.61 to −0.11, *p* = 0.005, I^2^ = 53%, Figure 3C). No significant differences were observed between two groups in nasal congestion score (SMD, −0.16; 95% CI, −0.37 to −0.14, *p* = 0.02, I^2^ = 36%, Figure 3D) and rhinorrhea scores (SMD, −0.21; 95% CI, −0.45 to −0.02, *p* = 0.08, I^2^ = 47%, Figure 3E).

##### 3.4.2.3 RQLQ

3 trials reported RQLQ (Meltzer et al., 2000; Nayak et al., 2002; Miao, 2020). There were no significant differences in RQLQ (SMD, −0.39; 95% CI, −0.79 to −0.02, *p* = 0.06, I^2^ = 83%, Figure 3F) compared with montelukast.

#### 3.4.3 Loratadine-montelukast vs. placebo

##### 3.4.3.1 TNSS

**FIGURE 4:**
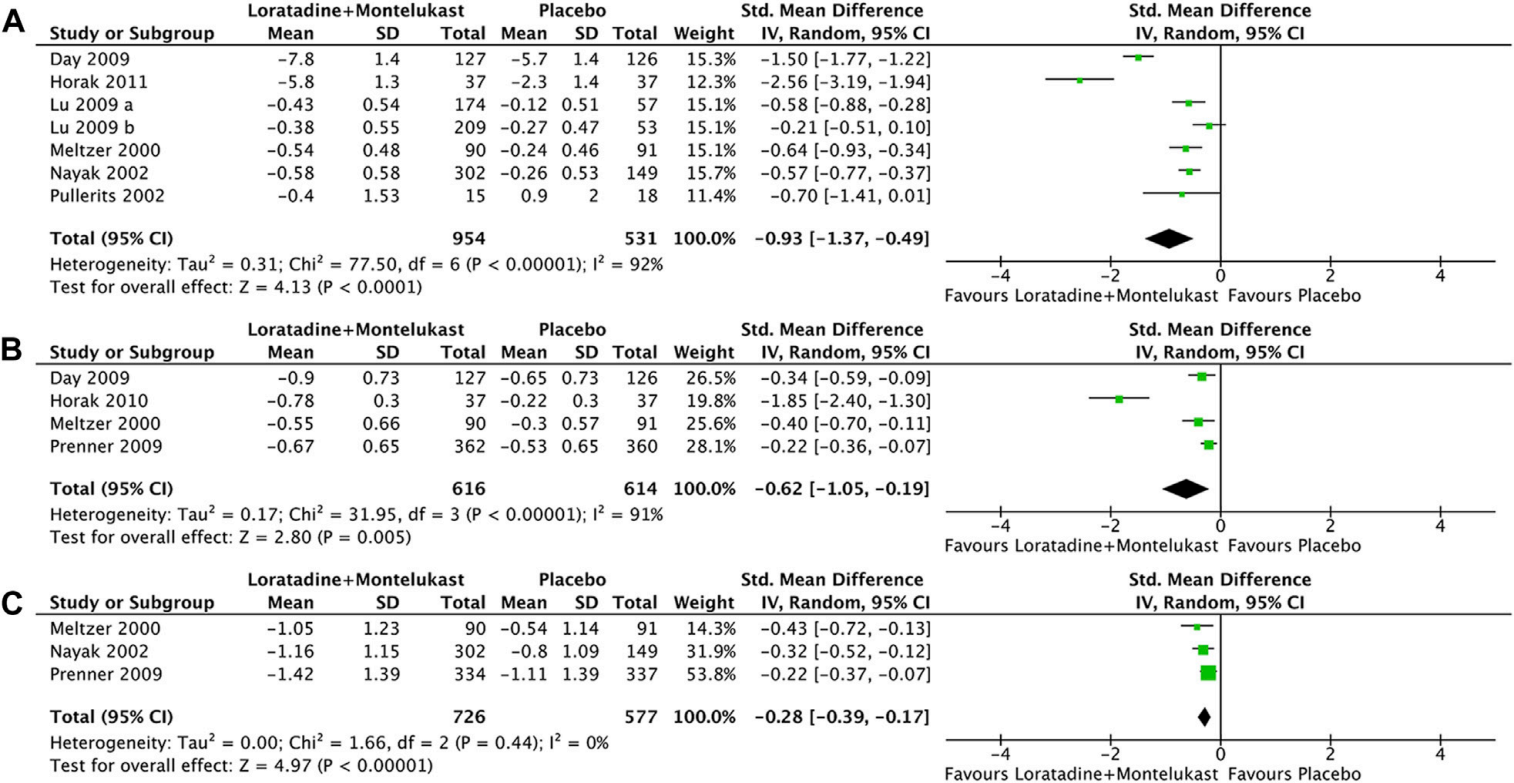
Forest plot for effect of loratadine-montelukast *versus* montelukast on total nasal symptom score **(A)**, nasal congestion **(B)**, and rhinoconjunctivitis quality-of-life questionnaires **(C)**.

Primary outcome was reported in 7 trials (Meltzer et al., 2000; Nayak et al., 2002; Pullerits et al., 2002; Day et al., 2009; Lu et al., 2009; Horak et al., 2010). Compared with placebo, loratadine-montelukast can significantly reduce TNSS (SMD, −0.93; 95% CI, −1.37 to −0.49, *p* < 0.00001, I^2^ = 92%, Figure 4A).

##### 3.4.3.2 Single nasal symptom score

Only 1 trial reported NCS, NIS, sneezing score, and rhinorrhea score simultaneously (Meltzer et al. 2000), and 3 trials reported NCS (Day et al., 2009; Prenner et al., 2009; Horak et al., 2010). Therefore, only NCS was analyzed in this meta-analysis. Compared with placebo, loratadine-montelukast can significantly reduce nasal congestion (SMD, −0.62; 95% CI, −1.05 to −0.19, *p* = 0.005, I^2^ = 91%, Figure 4B).

##### 3.4.3.3 RQLQ

There were 3 trials reported in RQLQ (Meltzer et al. 2000; Nayak et al. 2002; Prenner et al. 2009). This meta-analysis demonstrated a significant improvement in RQLQ (SMD, −0.28; 95% CI, −0.39 to −0.17, *p* < 0.00001, I^2^ = 0%, Figure 4C) in the loratadine-montelukast group compared with placebo.

#### 3.4.4 Additional analysis

The subgroup analysis was performed by dividing into adults or children group. Compared with loratadine monotherapy, loratadine-montelukast can significantly improve TNSS either in children subgroup (SMD, −0.81; 95% CI, −1.04 to −0.58, *p* < 0.00001, I^2^ = 0%, Supplementary Figure S1) or adult subgroup (SMD, −1.09; 95% CI, −1.52 to −0.65, *p* < 0.00001, I^2^ = 94%, Supplementary Figure S1). Compared with montelukast monotherapy, loratadine-montelukast can significantly improve TNSS either in children subgroup (SMD, −0.80; 95% CI, −1.07 to −0.52, *p* < 0.00001, I^2^ = 0%, Supplementary Figure S2) or in adult subgroup (SMD, −0.37; 95% CI, −0.59 to −0.15, *p* = 0.0009, I^2^ = 67%, Supplementary Figure S2). Another subgroup analysis was performed by dividing into English articles or Chinese articles. Compared with loratadine monotherapy, loratadine-montelukast can significantly improve TNSS either in English article subgroup (SMD, −0.33; 95% CI, −0.59 to −0.07, *p* = 0.01, I^2^ = 74%, Supplementary Figure S3) or Chinese article subgroup (SMD, −1.38; 95% CI, −1.85 to −0.92, *p* < 0.00001, I^2^ = 88%, Supplementary Figure S3). Compared with montelukast monotherapy, loratadine-montelukast can significantly improve TNSS either in English article subgroup (SMD, −0.33; 95% CI, −0.56 to −0.11, *p* = 0.004, I^2^ = 66%, Supplementary Figure S4) or Chinese article subgroup (SMD, −0.76; 95% CI, −1.00 to −0.53, *p* < 0.00001, I^2^ = 0%, Supplementary Figure S4).

#### 3.4.5 Assessment of sensitivity analysis

Sensitivity analysis was performed on the primary outcome measure by omitting one study at a time using a random-effects model. Sensitivity analysis results showed that the pooled result and heterogeneity had no significant change (Supplementary Figure S5).

#### 3.4.6 Adverse events

Adverse events were reported in 12 studies (Meltzer et al., 2000; Nayak et al., 2002; Day et al., 2009; Prenner et al., 2009; Horak et al., 2010; Gong, 2016; Lin, 2017; Bian and Cui, 2018; Li, 2019; Qiao, 2020; Zhang et al., 2021; Shi et al., 2022), 1 study reported no adverse event (Liu, Zeng, and Zeng, 2019), and adverse events were not reported in the remaining 10 studies (Pullerits et al., 2002; Hung et al., 2007; Xiao and Zhang, 2008; Huang et al., 2009; Lu et al., 2009; Yamamoto et al., 2012; Cai et al., 2017; Miao, 2020; Shen et al., 2020; Lu et al., 2022). 11 studies reported similar rates of adverse events between the intervention group and the control group (Meltzer et al., 2000; Nayak et al., 2002; Day et al., 2009; Prenner et al., 2009; Horak et al., 2010; Gong, 2016; Bian and Cui, 2018; Li, 2019; Qiao, 2020; Zhang et al., 2021; Shi et al., 2022). Only 1 reported that the incidence of adverse events in the intervention group was significantly lower than that in the control group (Lin, 2017). There were no serious adverse events in all studies. Among the 23 articles, 7 subjects did not complete the study. The most common adverse events were headache, dry mouth, and drowsiness. Some studies reported dizziness, rash, nausea, vomiting, abdominal pain, diarrhea, bloating, and other epigastric discomfort. Few adverse events were reported as fatigue, insomnia, feeling sluggish, upper respiratory tract infection, nervousness, and irritability.

### 3.5 Quality of studies and publication bias

**FIGURE 5:**
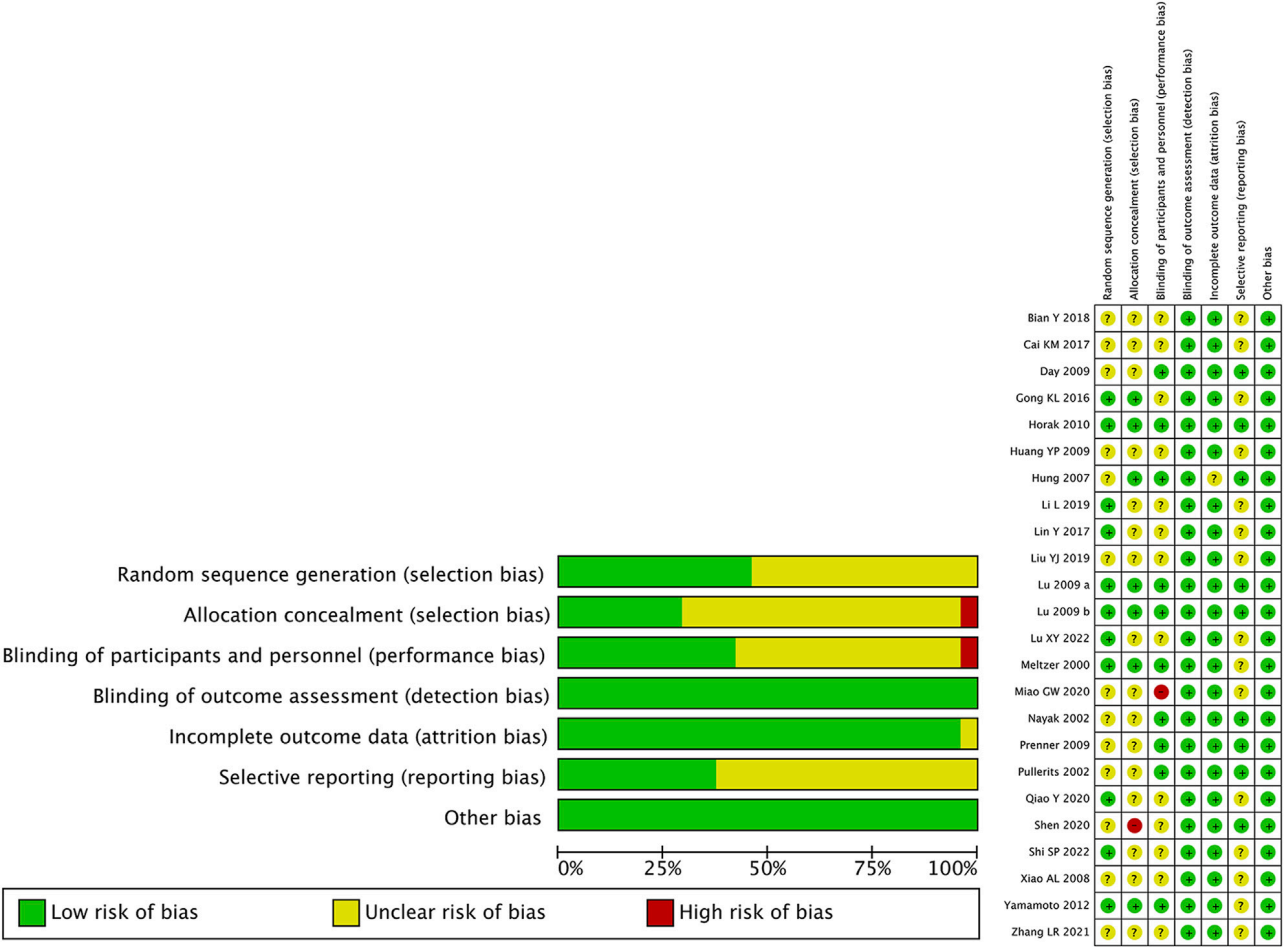
Summary risk bias of included studies.

We used the Cochrane Handbook for Systematic Reviews of Interventions as quality evaluation criteria to assess the risk of bias. Among these studies, 45.8% had a low risk of bias in random sequence generation (Meltzer et al., 2000; Lu et al., 2009; Horak et al., 2010; Yamamoto et al., 2012; Gong, 2016; Lin, 2017; Li, 2019; Qiao, 2020; Lu et al., 2022; Shi et al., 2022) and reported concrete method of randomization, 29.2% had low risk of bias (Meltzer et al., 2000; Hung et al., 2007; Lu et al., 2009; Horak et al., 2010; Yamamoto et al., 2012; Gong, 2016) and 4.2% had a high risk of bias (Shen et al., 2020) in allocation concealment, 41.7% had a low risk of bias (Meltzer et al., 2000; Nayak et al., 2002; Pullerits et al., 2002; Hung et al., 2007; Day et al., 2009; Lu et al., 2009; Prenner et al., 2009; Horak et al., 2010; Yamamoto et al., 2012) and 4.2% had high risk (Miao, 2020) of bias in the blinding of participants and personnel, 100% had a low risk of bias in the blinding of outcome assessment, 95.8% had a low risk of bias in incomplete outcome data (Meltzer et al., 2000; Nayak et al., 2002; Pullerits et al., 2002; Xiao and Zhang, 2008; Day et al., 2009; Huang et al., 2009; Lu et al., 2009; Prenner et al., 2009; Horak et al., 2010; Yamamoto et al., 2012; Gong, 2016; Cai et al., 2017; Lin, 2017; Bian and Cui, 2018; Li, 2019; Liu, Zeng, and Zeng, 2019; Miao, 2020; Qiao, 2020; Shen et al., 2020; Zhang et al., 2021; Lu et al., 2022; Shi et al., 2022), and 37.5% had a low risk of bias in selective reporting (Nayak et al., 2002; Pullerits et al., 2002; Hung et al., 2007; Day et al., 2009; Lu et al., 2009; Prenner et al., 2009; Horak et al., 2010; Shen et al., 2020). The summary risk bias was shown in Figure 5. The funnel plots show the asymmetry of the primary outcome, indicating there is publication bias (Supplementary Figure S6).

## 4 Discussion

In the present meta-analysis, we performed a comprehensive search of RCTs, and finally identified 23 studies with 4,902 participants. The pooled results showed that loratadine-montelukast was superior in the treatment of AR, when compared with loratadine monotherapy, montelukast monotherapy or placebo. Subgroup analysis showed that loratadine-montelukast is still associated with better alleviation of TNSS in both adults and children with AR. Finally, loratadine-montelukast did not increase side effects compared to monotherapy, and all patients tolerated combination therapy.

Histamine and cysteinyl leukotriene are common inflammatory mediators leading to the pathogenesis of AR (Cobanoğlu et al., 2013). These two mediators stimulate different receptors, including histamine receptors, CysLT1 receptor, and jointly participate in the early-phase response of AR. Nasal itching, sneezing and rhinorrhea in the early-phase response are mainly caused by sensory nerve stimulation symptoms caused by histamine, which can also cause nasal congestion to varying degrees (Bjermer et al., 2019). Loratadine is a commonly used long-acting antihistamine. It has an obvious competitive inhibition on histamine H1 receptor, and can significantly inhibit the release of leukotrienes and histamine by mast cells, and improve inflammation and allergic reactions by reducing capillary permeability (Haria, Fitton, and Peters, 1994). Nasal congestion is a symptom related to vasodilation and vascular leakage in the late-phase reaction of anaphylaxis (Thompson, Sardana, and Craig, 2013). Antihistamines cannot effectively control the symptoms of late-phase nasal congestion. Leukotrienes mainly participate in the late-phase reaction of allergic rhinitis, leading to persistent nasal congestion (Sin and Togias, 2011). Compared with histamine, leukotrienes cause a greater increase in nasal airway resistance. Leukotrienes stimulate the secretion of mucus by relaxing vascular smooth muscle, promoting chemotaxis and adhesion of eosinophils (Peters-Golden, Gleason, and Togias, 2006). Currently, clinically used anti-leukotriene drugs are divided into two categories, cysteinyl leukotriene receptor antagonists and leukotriene synthesis inhibitors (Lipworth, 1999; Steinke and Culp, 2007). Leukotriene receptor antagonists are non-hormonal anti-inflammatory drugs, which mainly play a role by competitively binding to cysteinyl leukotriene receptors and blocking the activity of cysteinyl leukotriene receptors (Peters-Golden, Gleason, and Togias, 2006). Montelukast is one of the representative drugs of CysLTs receptor antagonists with high selectivity. It can reduce the synthesis and release of inflammatory mediators in nasal mucosa caused by allergen stimulation, and control nasal congestion symptoms in AR (Philip et al., 2002; van Adelsberg et al., 2003). Due to the different targets of loratadine and montelukast in AR, therefore, the combination of them might yield additional benefits. The goal of this meta-analysis aims to provide this evidence.

In this study, based on the principles of Cochran’s systematic review, a meta-analysis of loratadine combined with montelukast in the treatment of AR was conducted. The control group included loratadine monotherapy, or montelukast monotherapy, or placebo. There are no restrictions on age, gender and duration of disease. The results of this meta-analysis showed that the efficacy of loratadine combined with montelukast in the treatment of AR was more significant than that of loratadine, montelukast or placebo. The primary outcome and secondary outcome helps to support this conclusion. But there was no difference in improving RQLQ, nasal congestion, and rhinorrhea between loratadine-montelukast and montelukast groups. The lack of finding a statistical difference in these individual symptoms may be related to the limited number of relevant articles. A combination of therapy for AR has been proposed several decades ago, however, it is hard to find the right combination of medications that provide additional therapy without increasing adverse effects (Greiwe and Bernstein, 2016). Some combination therapies have been suggested to increase therapeutic effects. Dockhorn et al. (Dockhorn et al., 1999) showed that the combined use of ipratropium bromide nasal spray and beclomethasone dipropionate nasal spray has better effects than either active agent in improving AR symptoms. A randomized, multicenter trial showed that the montelukast/levocetirizine group had greater improvement in nasal symptoms than the montelukast group (Kim et al., 2018). However, some combination fails to show additional therapeutic effects. Ratner et al. (Ratner et al., 1998) found that combination of fluticasone propionate nasal spray (FPNS) and loratadine has a similar treatment effect as FPNS monotherapy in almost all evaluations. LaForce et al. demonstrated that the combination of azelastine nasal spray and fexofenadine has the same treatment effect as azelastine nasal spray monotherapy in alleviating TNSS (LaForce et al., 2004). Andhale et al. (Andhale, Goel, and Nayak, 2016) found that compared monotherapy with montelukast or levocetirizine, their combination had no additional improvement in AR. Now, This meta-analysis demonstrates that the loratadine-montelukast combination significantly improves AR symptoms when compared to placebo and to loratadine or montelukast monotherapy. In terms of safety, the incidence of adverse reactions was similar among all groups. No serious adverse reactions were observed in the montelukast-loratadine group.

It is undeniable that this study has certain limitations. First, we only searched articles published in English, and Chinese. Second, the quality of the included articles was not high, and some articles did not mention blinding method and allocation concealment, especially in the Chinese articles. Third, moderate to high heterogeneity were found in the primary outcome. We performed the subgroup analyses to reduce heterogeneity, however, it was not significantly reduced.

In conclusion, loratadine combined with montelukast is superior to loratadine monotherapy, montelukast monotherapy or placebo in the improvement of AR symptoms. Therefore, we recommend that combination therapy of loratadine and montelukast as an option for patients with moderate-severe AR or patients with poor efficacy of single therapy.

## Data Availability

The original contributions presented in the study are included in the article/Supplementary Material, further inquiries can be directed to the corresponding author.

## Abbreviations

AR, allergic rhinitis; CNKI, China National Knowledge Infrastructure; TNSS, total nasal symptom scores; RQLQ, rhinoconjunctivitis quality of life questionnaires; RCTs, randomized controlled trials; NCS, nasal congestion score; NIS, nasal itching score; SMD, standardized mean difference; CI, confidence interval; FPNS, fluticasone propionate nasal spray.
