# Supplementary material for: A systematic review and meta-analysis of loratadine combined with montelukast for the treatment of allergic rhinitis
Source: Front Pharmacol. 2023 Oct 17;14:1287320. doi: 10.3389/fphar.2023.1287320 (PMC10616259; doi:10.3389/fphar.2023.1287320)
Supplement: Supplementary file 1 [file Table1.DOCX]

Table 1: Basic characteristics of subjects and treatments of trials

| Source | Country | Type of patients | Duration | Intervention | | | Control | | | Outcomes | Adverse event |
| --- | --- | --- | --- | --- | --- | --- | --- | --- | --- | --- | --- |
|  |  |  |  | Intervention method（Dose） | Population  （Male） | Mean age  (years ± SD) | Intervention method（Dose） | Population  （Male） | Mean age  (years ± SD) |  |  |
| Bian Y et.al 2018 | China | AR | 2 weeks | Loratadine-montelukast  (10mg-4mg, once/day) | 65(40) | 37.4±11.5 | Loratadine  (10mg, once/day) | 65(42) | 37.5±11.6 | TNSS | Headache, Dry mouth, Drowsiness |
| Cai KM 2017 | China | AR in children | 2 weeks | Loratadine-montelukast  (10/5mg-5/4mg, once/day) | 38(20) | 5.9±2.1 | Loratadine  (10/5mg, once/day) | 38(21) | 5.5±2.4 | VAS, Nasal Congestion score | Not reported |
| Day et.al 2009 | Canada | SAR | 8 hours | Loratadine-montelukast  (10mg-10mg, once/day) | 127(49) | 33.6±11.3 | Placebo (once/day) | 126(51) | 33.4±10.6 | TNSS, Nasal Congestion score | abdominal distention,  abdominal pain,  epigastric discomfort,  headache,  somnolence,  nausea,  hypoesthesia,  disorientation,  urticaria |
| Gong KL 2016 | China | AR | 2 weeks | Loratadine-montelukast  (10mg-4mg, once/day) | 42(30) | 40.34±3.29 | Loratadine  (10 mg, once/day) | 41(29) | 40.13±3.02 | TNSS  RQLQ | Headache dry mouth drowsiness |
| Horak et. al 2010 | Austria | SAR | 4 hours | Loratadine-montelukast  (10mg-10mg) | 37(17) | 25 | Placebo | 37(15) | 26.5 | TNSS, Nasal Congestion score | mild upper respiratory tract infection, severe knee fracture |
| Huang YP et.al 2009 | China | AR in children | 3 weeks | Loratadine-montelukast  (10/5mg-5/4mg, once/day) | 50(27) | 9.45±3.97 | Loratadine  (10/5 mg, once/day) | 50(24) | 9.23±4.46 | TNSS | Not reported |
|  |  |  |  |  |  |  | montelukast  (5/4mg, once/day) | 50(28) | 9.13±3.78 |  |  |
| Hung et.al 2007 | China Taiwan | PAR in children | 8weeks | Loratadine-montelukast  (5mg-5mg) | 11(6) | 8.45±1.51 | Loratadine  (5mg) | 11(5) | 7.81±1.94 | TNSS | Not reported |
| Li L 2019 | China | AR | 2 weeks | Loratadine-montelukast  (10mg-4mg, once/day) | 40(23) | 43.51±8.37 | Loratadine  (10mg, once/day) | 40(24) | 43.46±8.64 | TNSS | headache, dry mouth, drowsiness |
| Lin Y 2017 | China | AR | 2 weeks | Loratadine-montelukast  (10mg-4mg, once/day) | 40(19) | 36.4±2.8 | Loratadine  (10mg, once/day) | 40(22) | 37.2±2.5 | TNSS  RQLQ | headache, dry mouth, drowsiness |
| Liu YJ et.al 2019 | China | AR | 1 month | Loratadine-montelukast  (8.8mg-10mg, once/day) | 52(24) | Not reported | Loratadine  (8.8mg, once/day) | 52(27) | Not reported | VAS  Nasal Congestion, Itching, Sneezing, Rhinorrhea score | No adverse reactions |
| Lu et.al 2009 | USA | SAR | 2 weeks | Loratadine-montelukast  (10mg-10mg) | 174 | 34.0±12.7 | Loratadine  (10mg) | 115 | 34.8±12.4 | TNSS | Not reported |
|  |  |  |  |  |  |  | Montelukast (10mg) | 111 | 35.6±13.1 |  |  |
|  |  |  |  |  |  |  | Placebo | 57 | 35.1±13.8 |  |  |
|  |  | SAR and asthma | 2 weeks | Loratadine-montelukast (10mg-10mg) | 209 | 32.8±12.6 | Loratadine  (10mg) | 162 | 30.6±10.9 | TNSS | Not reported |
|  |  |  |  |  |  |  | Montelukast (10mg) | 103 | 31.1±13.1 |  |  |
|  |  |  |  |  |  |  | Placebo | 53 | 33.6±13.5 |  |  |
| Lu XY et.al 2022 | China | AR in children | 3 months | Loratadine-montelukast + triamcinolone acetonide  (10/5mg-5mg, once/day) | 42(26) | 11.19±2.12 | Loratadine + triamcinolone acetonide  (10/5mg, once/day) | 42(23) | 11.81±2.18 | Nasal Congestion, Itching, Sneezing, Rhinorrhea score | Not reported |
| Meltzer et.al 2000 | USA | SAR | 2 weeks | Loratadine-montelukast (10mg-10mg) | 90(44) | 37 | Loratadine  (10mg) | 92(43) | 34.5 | TNSS, Nasal Congestion, Itching, Sneezing, Rhinorrhea score, RQLQ | Headache, upper respiratory tract infection |
|  |  |  |  |  |  |  | Montelukast (10mg) | 95(40) | 33 |  |  |
|  |  |  |  |  |  |  | Montelukast (20mg) | 90(33) | 34.5 |  |  |
|  |  |  |  |  |  |  | Placebo | 91(45) | 33 |  |  |
| Miao GW 2020 | China | AR | 2 weeks | Loratadine-montelukast (4mg-10mg, once/day) | 40(25) | 36.49±4.52 | Montelukast (10mg, once/day) | 40(24) | 36.58±4.71 | TNSS  RQLQ | Not reported |
| Nayak et.al 2002 | USA | SAR | 2 weeks | Loratadine-montelukast (10mg-10mg) | 302(94) | 38±13 | Loratadine  (10mg) | 301(110) | 37 ± 13 | TNSS, Nasal Congestion, Itching, Sneezing, Rhinorrhea score, RQLQ | Headache, dry mouth, asthenia, fatigue, tachycardia, prurit |
|  |  |  |  |  |  |  | Montelukast (10 mg) | 155(53) | 35 ± 11 |  |  |
|  |  |  |  |  |  |  | Placebo | 149(63) | 37 ± 13 |  |  |
| Prenner et.al 2009 | USA | SAR | 15 days | Loratadine-montelukast (10mg-10mg) | 363(126) | 26±7 | Placebo | 363(144) | 22±6 | Nasal Congestion score, RQLQ | Dry mouth, Nausea, Headache, Insomnia, Vertigo, Irritability, Psychomotor hyperactivity, Tremor, Nervousness, Restlessness |
| Pullerits et.al 2002 | Sweden | SAR | 2 months | Loratadine-montelukast (10mg-10mg) | 15(6) | 30.1±9.9 | Montelukast (10mg) | 16(10) | 28.3±8.0 | TNSS | Not reported |
|  |  |  |  |  |  |  | Placebo | 18(13) | 29.8±10.4 |  |  |
| Qiao Y 2020 | China | AR | 2 weeks | Loratadine-montelukast  (8.8mg-10mg, once/day) | 51(30) | 42.39±5.46 | Loratadine  (8.8mg, once/day) | 51(28) | 42.57±5.53 | Nasal Congestion, Itching, Sneezing, Rhinorrhea score | dry mouth, drowsiness, dizziness, rash |
| Shen et.al 2020 | China | Patients with AR in sneezing group | 8 weeks | Loratadine-montelukast + mometasone furoate (10 mg/10 mg) | 9 | Not reported | Loratadine + mometasone furoate | 8 | Not reported | TNSS | Not reported |
|  |  |  |  |  |  |  | montelukast +mometasone furoate | 8 |  |  |  |
|  |  | Patients with AR in nasal congestion  group | 8 weeks | Loratadine-montelukast + mometasone furoate (10 mg/10 mg) | 10 | Not reported | Loratadine + mometasone furoate | 9 | Not reported | TNSS | Not reported |
|  |  |  |  |  |  |  | montelukast +mometasone furoate | 9 |  |  |  |
|  |  | Patients with AR in sneezing and nasal congestion  group | 8 weeks | Loratadine-montelukast + mometasone furoate (10 mg/10 mg) | 10 | Not reported | Loratadine + mometasone furoate | 9 | Not reported | TNSS | Not reported |
|  |  |  |  |  |  |  | montelukast +mometasone furoate | 10 |  |  |  |
| Shi SP et.al 2022 | China | AR | 2 weeks | Loratadine-montelukast (10mg-10mg, once/day) | 37(21) | 44.23±3.18 | Loratadine  (10mg, once/day) | 37(22) | 43.96±3.05 | Nasal Congestion, Itching, Sneezing, Rhinorrhea score | diarrhea, rash, dizziness, headache, nausea and vomiting |
| Xiao AL et.al 2008 | China | AR in children | 2 weeks | Loratadine-montelukast  (10/5mg-5/4mg, once/day) | 60(32) | 8.52±3.2 | Loratadine  (10/5mg, once/day) | 60(28) | 8.45±3.27 | TNSS | Not reported |
|  |  |  |  |  |  |  | montelukast  (5/4mg, once/day) | 60(35) | 8.33±3.16 |  |  |
| Yamamoto et.al 2012 | Japan | SAR | 50 days | Loratadine-montelukast (10mg-10mg) | 21(7) | 26.7±2.4 | Montelukast-placebo (10mg) | 21(10) | 26.4±2.2 | TNSS | Not reported |
| Zhang LR et.al 2021 | China | AR | 2 weeks | Loratadine-montelukast  (10mg-5mg, once/day) | 41(23) | 41.2±4.59 | Loratadine  (10mg, once/day) | 41(24) | 42.16±4.72 | Nasal Congestion, Itching, Sneezing, Rhinorrhea score | headache, dry mouth, drowsiness |

RCT: randomized controlled trial; AR: allergic rhinitis; TNSS: total nasal symptom scores; SAR: seasonal allergic rhinitis; PAR: perennial allergic rhinitis; RQLQ: rhinoconjunctivitis quality of life questionnaires; VAS: visual analogue scale
