# Supplementary material for: A systematic review and meta-analysis of loratadine combined with montelukast for the treatment of allergic rhinitis
Source: Front Pharmacol. 2023 Oct 17;14:1287320. doi: 10.3389/fphar.2023.1287320 (PMC10616259; doi:10.3389/fphar.2023.1287320)
Supplement: Supplementary file 2 [file DataSheet1.PDF]

A Systematic review and meta-analysis of loratadine combined with montelukast for the treatment of allergic rhinitis

Supplementary Online Content

Table 1. Search strategy in PubMed

PubMed		
1	"Rhinitis, Allergic"[Mesh]	23101
2	((Allergic Rhinitides[Title/Abstract]) OR (Rhinitides, Allergic[Title/Abstract])) OR (Allergic Rhinitis[Title/Abstract])	26462
3	"Loratadine"[Mesh]	1244
4	((((((4-(8-Chloro-5,6-dihydro-11H-benzo(5,6)cyclohepta(1,2-b)pyridin-11-ylidene)-1-piperidinecarboxylic Acid Ethyl Ester[Title/Abstract]) OR (Claritin[Title/Abstract])) OR (Sch-29851[Title/Abstract])) OR (Sch 29851[Title/Abstract])) OR (Sch29851[Title/Abstract])) OR (Alavert[Title/Abstract])) OR (Clarium[Title/Abstract])	71
5	"montelukast" [Supplementary Concept]	1895
6	(((((1-((((1R)-1-(3-((E)-2-(7-chloro-2-quinolinyl)ethenyl)phenyl)-3-(2-(1-hydroxy-1-methylethyl)phenyl)propyl)thio)methyl)-cyclopropaneacetic acid[Title/Abstract]) OR (MK 0476[Title/Abstract])) OR (MK-0476[Title/Abstract])) OR (Singulair[Title/Abstract])) OR (montelukast sodium[Title/Abstract])) OR (sodium 1-(((1-(3-(2-(7-chloro-2-quinolinyl)ethenyl)phenyl)-3-(2-(1-hydroxy-1-methylethyl)phenyl)propyl)thio)methyl)cyclopropylacetate[Title/Abstract])	286
7	Randomized controlled trial OR Controlled clinical trial OR Randomized OR Placebo OR Randomly	1731553
8	(1 OR 2) and (3 OR 4) and (5 OR 6) and (7)	41

Study or Subgroup	Loratadine + Montelukast Mean	SD	Total	Loratadine Mean	SD	Total	Weight	Std. Mean Difference IV, Random, 95% CI
**8.1.1 AR in children**								
Cai KM 2017	−4.8	2.39	38	−2.1	2.41	38	6.6%	−1.11 [−1.60, −0.63]
Huang YP 2009	−1.77	0.58	50	−1.34	0.63	50	6.8%	−0.70 [−1.11, −0.30]
Hung 2007	−2.92	1	11	−2.36	0.9	11	5.2%	−0.57 [−1.42, 0.29]
Xiao AL 2008	−1.8	0.51	60	−1.35	0.64	60	6.9%	−0.77 [−1.14, −0.40]
**Subtotal (95% CI)**			**159**			**159**	**25.6%**	**−0.81 [−1.04, −0.58]**
Heterogeneity: Tau² = 0.00; Chi² = 2.11, df = 3 (P = 0.55); I² = 0%								
Test for overall effect: Z = 6.94 (P < 0.00001)								
**8.1.2 AR in adults**								
Bian Y 2018	−3.9	2.48	65	−2.3	2.36	65	7.0%	−0.66 [−1.01, −0.30]
Gong KL 2016	−2.19	0.72	42	−0.92	0.66	41	6.5%	−1.82 [−2.34, −1.31]
Li L 2019	−1.78	0.2	40	−1.2	0.32	40	6.3%	−2.15 [−2.71, −1.60]
Lin Y 2017	−2.13	0.49	40	−0.88	0.43	40	6.1%	−2.69 [−3.30, −2.07]
Liu YJ 2019	−5.49	1.53	52	−3.37	1.44	52	6.8%	−1.42 [−1.85, −0.98]
Lu 2009 a	−0.43	0.54	174	−0.42	0.52	115	7.3%	−0.02 [−0.25, 0.22]
Lu 2009 b	−0.38	0.55	209	−0.29	0.55	162	7.3%	−0.16 [−0.37, 0.04]
Meltzer 2000	−0.54	0.48	90	−0.32	1.54	92	7.2%	−0.19 [−0.48, 0.10]
Nayak 2002	−0.58	0.58	302	−0.52	0.53	301	7.4%	−0.11 [−0.27, 0.05]
Shen 2020 a	−0.96	0.07	9	−0.93	0.06	8	4.8%	−0.43 [−1.40, 0.53]
Shen 2020 b	−0.98	0.05	10	−0.56	0.21	9	3.6%	−2.70 [−4.02, −1.38]
Shen 2020 c	−1.01	0.02	10	−0.82	0.13	9	4.2%	−2.01 [−3.16, −0.86]
**Subtotal (95% CI)**			**1043**			**934**	**74.4%**	**−1.09 [−1.52, −0.65]**
Heterogeneity: Tau² = 0.50; Chi² = 193.47, df = 11 (P < 0.00001); I² = 94%								
Test for overall effect: Z = 4.89 (P < 0.00001)								
**Total (95% CI)**			**1202**			**1093**	**100.0%**	**−1.00 [−1.35, −0.65]**
Heterogeneity: Tau² = 0.43; Chi² = 205.90, df = 15 (P < 0.00001); I² = 93%								
Test for overall effect: Z = 5.60 (P < 0.00001)								
Test for subgroup differences: Chi² = 1.18, df = 1 (P = 0.28), I² = 15.3%								

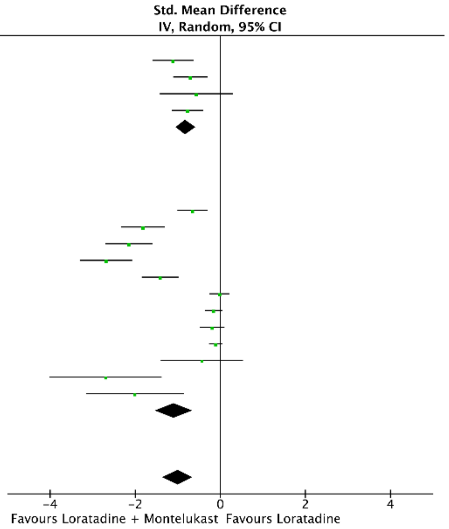

Fig. 1: Loratadine + Montelukast vs Loratadine for total nasal symptom score in children or adult.

Study or Subgroup	Loratadine + Montelukast Mean	SD	Total	Montelukast Mean	SD	Total	Weight	Std. Mean Difference IV, Random, 95% CI
**8.2.1 AR in children**								
Huang YP 2009	−1.77	0.58	50	−1.33	0.57	50	8.9%	−0.76 [−1.17, −0.35]
Xiao AL 2008	−1.8	0.51	60	−1.35	0.57	60	9.3%	−0.83 [−1.20, −0.45]
**Subtotal (95% CI)**			**110**			**110**	**18.2%**	**−0.80 [−1.07, −0.52]**
Heterogeneity: Tau² = 0.00; Chi² = 0.06, df = 1 (P = 0.81); I² = 0%								
Test for overall effect: Z = 5.67 (P < 0.00001)								
**8.2.2 AR in adults**								
Lu 2009 a	−0.43	0.54	174	−0.31	0.51	111	11.2%	−0.23 [−0.47, 0.01]
Lu 2009 b	−0.38	0.55	209	−0.34	0.52	103	11.2%	−0.07 [−0.31, 0.16]
Meltzer 2000 a	−0.54	0.48	45	−0.39	0.45	95	9.5%	−0.32 [−0.68, 0.03]
Meltzer 2000 b	−0.54	0.48	45	−0.31	1.52	90	9.5%	−0.18 [−0.54, 0.18]
Miao GW 2020	−1.78	0.62	40	−1.33	0.69	40	8.3%	−0.68 [−1.13, −0.23]
Nayak 2002	−0.58	0.58	302	−0.48	0.54	155	11.7%	−0.18 [−0.37, 0.02]
Pullerits 2002	−0.4	1.53	15	0.3	1.9	16	5.4%	−0.39 [−1.11, 0.32]
Shen 2020 a	−0.96	0.07	9	−0.69	0.13	8	2.1%	−2.50 [−3.85, −1.15]
Shen 2020 b	−0.98	0.05	10	−0.99	0.04	9	4.0%	0.21 [−0.69, 1.11]
Shen 2020 c	−1.01	0.02	10	−0.81	0.12	10	2.7%	−2.23 [−3.39, −1.06]
Yamamoto 2012	−82.3	52.11	21	−56.2	42.4	21	6.3%	−0.54 [−1.16, 0.08]
**Subtotal (95% CI)**			**880**			**658**	**81.8%**	**−0.37 [−0.59, −0.15]**
Heterogeneity: Tau² = 0.07; Chi² = 30.20, df = 10 (P = 0.0008); I² = 67%								
Test for overall effect: Z = 3.34 (P = 0.0009)								
**Total (95% CI)**			**990**			**768**	**100.0%**	**−0.46 [−0.68, −0.25]**
Heterogeneity: Tau² = 0.09; Chi² = 43.58, df = 12 (P < 0.0001); I² = 72%								
Test for overall effect: Z = 4.20 (P < 0.0001)								
Test for subgroup differences: Chi² = 5.62, df = 1 (P = 0.02), I² = 82.2%								

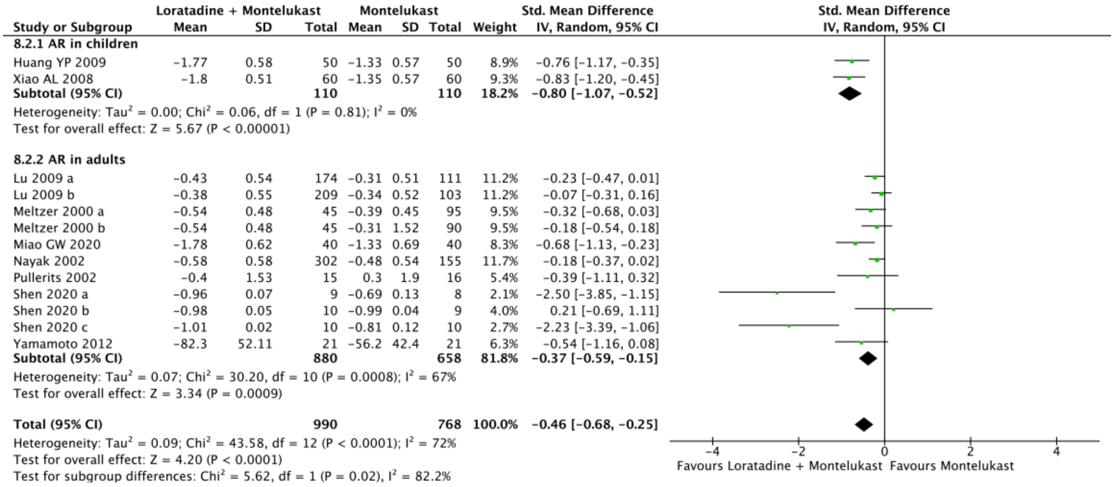

Fig. 2: Loratadine + Montelukast vs Montelukast for total nasal symptom score in children or adult.

Study or Subgroup	Loratadine + Montelukast Mean	SD	Total	Loratadine Mean	SD	Total	Weight	Std. Mean Difference IV, Random, 95% CI
**7.1.1 English articles**								
Bian Y 2018	−2.92	1	11	−2.36	0.9	11	5.2%	−0.57 [−1.42, 0.29]
Cai KM 2017	−0.43	0.54	174	−0.42	0.52	115	7.3%	−0.02 [−0.25, 0.22]
Gong KL 2016	−0.38	0.55	209	−0.29	0.55	162	7.3%	−0.16 [−0.37, 0.04]
Huang YP 2009	−0.54	0.48	90	−0.32	1.54	92	7.2%	−0.19 [−0.48, 0.10]
Li L 2019	−0.58	0.58	302	−0.52	0.53	301	7.4%	−0.11 [−0.27, 0.05]
Lin Y 2017	−0.96	0.07	9	−0.93	0.06	8	4.8%	−0.43 [−1.40, 0.53]
Liu YJ 2019	−0.98	0.05	10	−0.56	0.21	9	3.6%	−2.70 [−4.02, −1.38]
Xiao AL 2008	−1.01	0.02	10	−0.82	0.13	9	4.2%	−2.01 [−3.16, −0.86]
**Subtotal (95% CI)**			**815**			**707**	**47.0%**	**−0.33 [−0.59, −0.07]**
Heterogeneity: $Tau^2 = 0.08$; $Chi^2 = 27.15$, df = 7 (P = 0.0003); $I^2 = 74\%$								
Test for overall effect: Z = 2.50 (P = 0.01)								
**7.1.2 Chinese articles**								
Hung 2007	−3.9	2.48	65	−2.3	2.36	65	7.0%	−0.66 [−1.01, −0.30]
Lu 2009 a	−4.8	2.39	38	−2.1	2.41	38	6.6%	−1.11 [−1.60, −0.63]
Lu 2009 b	−2.19	0.72	42	−0.92	0.66	41	6.5%	−1.82 [−2.34, −1.31]
Meltzer 2000	−1.77	0.58	50	−1.34	0.63	50	6.8%	−0.70 [−1.11, −0.30]
Nayak 2002	−1.78	0.2	40	−1.2	0.32	40	6.3%	−2.15 [−2.71, −1.60]
Shen 2020 a	−2.13	0.49	40	−0.88	0.43	40	6.1%	−2.69 [−3.30, −2.07]
Shen 2020 b	−5.49	1.53	52	−3.37	1.44	52	6.8%	−1.42 [−1.85, −0.98]
Shen 2020 c	−1.8	0.51	60	−1.35	0.64	60	6.9%	−0.77 [−1.14, −0.40]
**Subtotal (95% CI)**			**387**			**386**	**53.0%**	**−1.38 [−1.85, −0.92]**
Heterogeneity: $Tau^2 = 0.40$; $Chi^2 = 60.51$, df = 7 (P < 0.00001); $I^2 = 88\%$								
Test for overall effect: Z = 5.80 (P < 0.00001)								
**Total (95% CI)**			**1202**			**1093**	**100.0%**	**−1.00 [−1.35, −0.65]**
Heterogeneity: $Tau^2 = 0.43$; $Chi^2 = 205.90$, df = 15 (P < 0.00001); $I^2 = 93\%$								
Test for overall effect: Z = 5.60 (P < 0.00001)								
Test for subgroup differences: $Chi^2 = 14.82$, df = 1 (P = 0.0001), $I^2 = 93.3\%$								

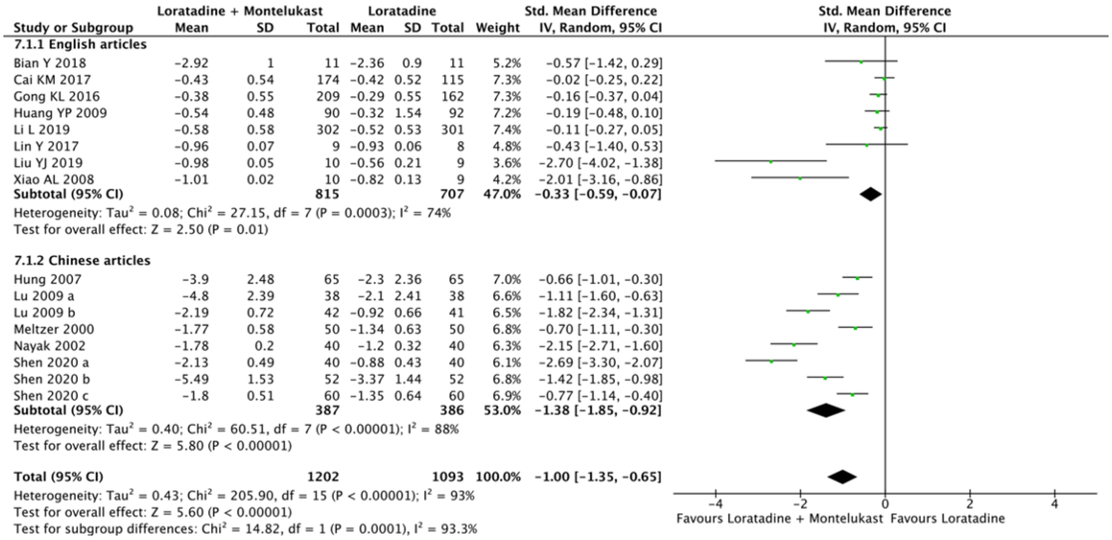

Fig. 3: Loratadine + Montelukast vs Loratadine for total nasal symptom score published in Chinese articles or English articles.

Study or Subgroup	Loratadine + Montelukast Mean	SD	Total	Montelukast Mean	SD	Total	Weight	Std. Mean Difference IV, Random, 95% CI
**7.2.1 English articles**								
Lu 2009 a	−0.43	0.54	174	−0.31	0.51	111	11.2%	−0.23 [−0.47, 0.01]
Lu 2009 b	−0.38	0.55	209	−0.34	0.52	103	11.2%	−0.07 [−0.31, 0.16]
Meltzer 2000 a	−0.54	0.48	45	−0.39	0.45	95	9.5%	−0.32 [−0.68, 0.03]
Meltzer 2000 b	−0.54	0.48	45	−0.31	1.52	90	9.5%	−0.18 [−0.54, 0.18]
Nayak 2002	−0.58	0.58	302	−0.48	0.54	155	11.7%	−0.18 [−0.37, 0.02]
Pullerits 2002	−0.4	1.53	15	0.3	1.9	16	5.4%	−0.39 [−1.11, 0.32]
Shen 2020 a	−0.96	0.07	9	−0.69	0.13	8	2.1%	−2.50 [−3.85, −1.15]
Shen 2020 b	−0.98	0.05	10	−0.99	0.04	9	4.0%	0.21 [−0.69, 1.11]
Shen 2020 c	−1.01	0.02	10	−0.81	0.12	10	2.7%	−2.23 [−3.39, −1.06]
Yamamoto 2012	−82.3	52.11	21	−56.2	42.4	21	6.3%	−0.54 [−1.16, 0.08]
**Subtotal (95% CI)**			**840**			**618**	**73.6%**	**−0.33 [−0.56, −0.11]**
Heterogeneity: $Tau^2 = 0.07$; $Chi^2 = 26.48$, df = 9 (P = 0.002); $I^2 = 66\%$								
Test for overall effect: Z = 2.90 (P = 0.004)								
**7.2.2 Chinese articles**								
Huang YP 2009	−1.77	0.58	50	−1.33	0.57	50	8.9%	−0.76 [−1.17, −0.35]
Miao GW 2020	−1.78	0.62	40	−1.33	0.69	40	8.3%	−0.68 [−1.13, −0.23]
Xiao AL 2008	−1.8	0.51	60	−1.35	0.57	60	9.3%	−0.83 [−1.20, −0.45]
**Subtotal (95% CI)**			**150**			**150**	**26.4%**	**−0.76 [−1.00, −0.53]**
Heterogeneity: $Tau^2 = 0.00$; $Chi^2 = 0.24$, df = 2 (P = 0.89); $I^2 = 0\%$								
Test for overall effect: Z = 6.38 (P < 0.00001)								
**Total (95% CI)**			**990**			**768**	**100.0%**	**−0.46 [−0.68, −0.25]**
Heterogeneity: $Tau^2 = 0.09$; $Chi^2 = 43.58$, df = 12 (P < 0.0001); $I^2 = 72\%$								
Test for overall effect: Z = 4.20 (P < 0.0001)								
Test for subgroup differences: $Chi^2 = 6.77$, df = 1 (P = 0.009), $I^2 = 85.2\%$								

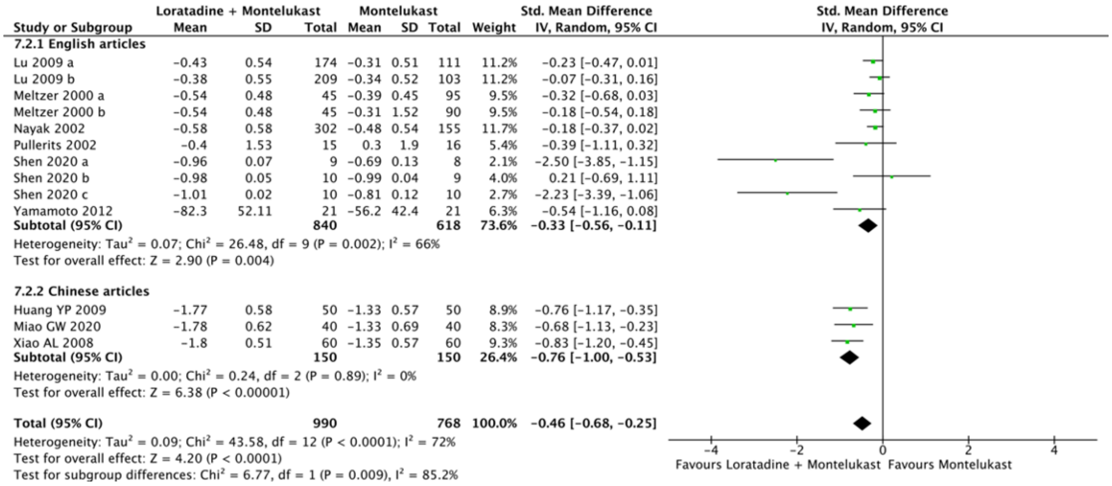

Fig. 4: Loratadine + Montelukast vs Montelukast for total nasal symptom score in English and Chinese articles subgroup

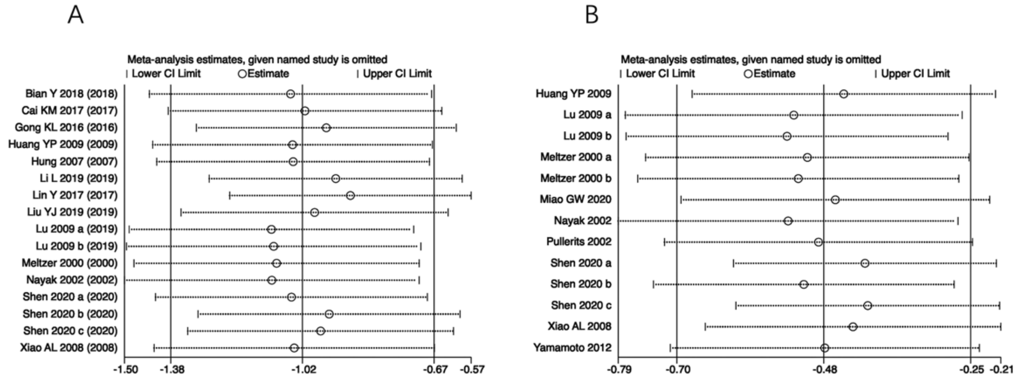

Fig. 5 Sensitivity analysis for total nasal symptom score. loratadine-montelukast versus loratadine (A), loratadine-montelukast versus montelukast (B)

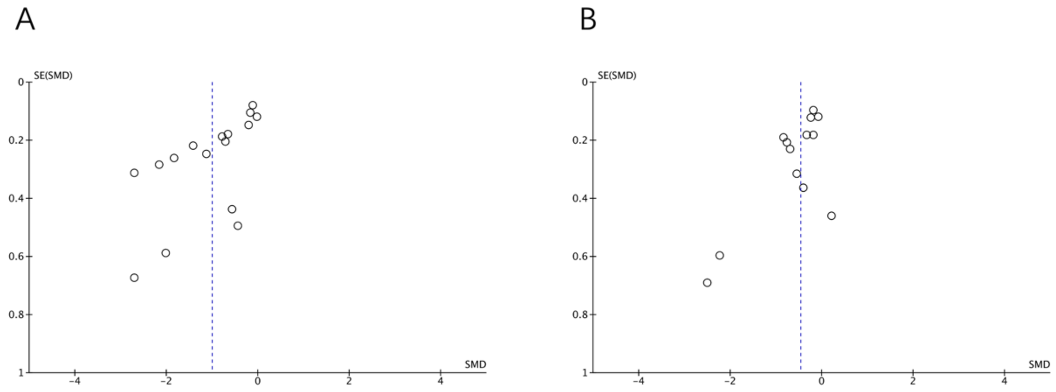

Fig. 6 Funnel plot for total nasal symptom score. loratadine-montelukast versus loratadine (A), loratadine-montelukast versus montelukast (B)
